# Differential responses of the seed germination of three functional groups to low temperature and darkness in a typical steppe, Northern China

**DOI:** 10.7717/peerj.14485

**Published:** 2022-12-01

**Authors:** Mengzhou Liu, Ning Qiao, Bing Zhang, Fengying Liu, Yuan Miao, Ji Chen, Yanfeng Sun, Peng Wang, Dong Wang

**Affiliations:** 1 College of Geography and Environmental Science, Henan University, Kaifeng, China; 2 International Joint Research Laboratory of Global Change Ecology, School of Life Sciences, Henan University, Kaifeng, China; 3 Institute of Microbial Engineering, Laboratory of Bioresource and Applied Microbiology, School of Life Sciences, Henan University, Kaifeng, China; 4 Department of Agroecology, Aarhus University, Tjele, Denmark; 5 iCLIMATE Interdisciplinary Centre for Climate Change, Aarhus University, Roskilde, Denmark; 6 Aarhus University Centre for Circular Bioeconomy, Aarhus University, Tjele, Denmark; 7 State Key Laboratory of Cotton Biology, School of Life Sciences, Henan University, Kaifeng, China, Kaifeng, China; 8 Hanzhong Urban Planning and Architectural Design Institute, Hanzhong, China

**Keywords:** Darkness, Germination percentage, Global change, Plant diversity, semiarid region

## Abstract

Seed germination is a key stage in the life history of plants, which has a crucial effect on plant community structure. Climate change has substantially altered the surface soil temperature and light availability, which can affect seed germination. However, whether the seed germination of different functional groups is affected by the interactions of light and temperature remains unclear. Under laboratory conditions, we examined the effects of low temperature and darkness, as well as their interaction, on the seed germination of 16 species belonging to three plant functional groups (annual and biennials, perennial grasses, and perennial forbs) in a typical steppe, Northern China. We found that low temperature had a significant negative effect on seed germination of all species. Low temperature significantly decreased the final germination percentage and germinative force of the three plant functional groups, and the germination duration of perennial grasses. Darkness significantly decreased the germinative force of perennial forbs and total seeds, and the germination duration of perennial grasses. The interactive effects of light and temperature on the seed final germination percentage and germinative force of perennial grass indicated that darkness strengthened the inhibitory effect of low temperature on the seed germination of the grass functional group. Our study indicate that the seed germination of different plant functional groups varied greatly in response to changing environmental conditions. Our results suggest that future climate change could alter the regeneration and species composition of plant communities through changing seed germination.

## Introduction

Seed germination is the initial stage of plant growth, and it affects the development and reproduction of individual plants, as well as the structure and composition of the plant community (Hoyle et al., 2014; Zhang et al., 2020). Compared with the vulnerability of seedlings, seeds are highly tolerant to environmental stress (Chen et al., 2019). The establishment of seedlings likely depends on the response of seed germination to the environment. Many plants have dormancy mechanisms to prevent germination, and seeds will break the seed coat and protrude the radicle until the conditions are suitable for seed germination and seedling growth (Miransari & Smith, 2014; Lai et al., 2019). Germination percentage and germination time determine the timing and location of seedling establishment, and affect species coexistence and plant community development (Tobe, Zhang & Omasa, 2005; Zhang et al., 2020). There is thus a need to identify the factors that affect the seed germination percentage and germination time.

Temperature and light have critically important effects on seed germination (El-Keblawy, 2017; Chen et al., 2019). Temperature is essential for breaking seed dormancy and inducing seed germination, as it stimulates enzyme activity in plant seeds, leading to the rupture of the seed coat, and enhances water permeability (Tabatabaei, 2015). The response of seed germination to temperature is often characterized by a parabolic relationship (Chen et al., 2019). There is an optimal temperature for seed germination, and temperatures above or below the optimum can inhibit seed germination (Durr et al., 2015). Ongoing global climate warming has not only resulted in a gradual increase in soil temperature but also led to shorter winters and the melting of snowpack in early spring (Walck et al., 2011). The lack of insulation from snow can lead to changes in soil and litter temperature, which can disrupt the regeneration of plants and alter the adaptive ranges of species due to frost exposure (Walck et al., 2011). Most studies have focused on clarifying the impact of higher temperatures on seed germination (Ronnenberg et al., 2007; Durr et al., 2015; Hadi et al., 2018). There is thus a need to study the effect of low temperature on seed germination to evaluate the responses of the community composition and structure to climate change.

Light also plays an important role in seed germination (Baskin & Baskin, 1998). Light can increase the content and activity of some enzymes in seeds and promote seed germination (Shahverdi et al., 2019). Light perceived by plants can be converted into internal signals that result in endogenous phytohormone responses (Seo et al., 2009). However, the seed germination of some plants is not light sensitive. For example, the seed germination of *Caragana korshinskii*, which grows on the dunes of Central Asia, shows no response to light (Zeng et al., 2003). The response of seeds to light is a mechanism that germination occurs under conditions conducive to seedling growth (Wang et al., 2014). Seedlings can be established to meet their own growth and nutritional requirements through photosynthesis (El-Keblawy, 2017). In environments where seeds may be buried in deep soil, covered by litter, or sheltered by caregivers, light is an important factor in determining the locations the most suitable for seedling establishment after germination (Wang et al., 2014; El-Keblawy, 2017). There is thus a need to study the response of grassland communities to changes in light. Although the effects of temperature and light on seed germination have been extensively studied, how the interaction between low temperature and darkness affects seed germination remains unclear.

The responses of the germination rate of different plant functional groups to nutrients and light vary under global change (Wang et al., 2014). Plant functional group is the group of plant species that share key functional traits, have similar response mechanisms to specific environmental factors, and have similar effects on the main ecosystem processes (Li et al., 2017; Su et al., 2018; Chen et al., 2020; Li et al., 2021). Therefore, the various responses of seed germination to light and temperature in plant communities may be related to the identity of plant functional groups. In a previous study examining the early succession of Mongolian steppe after drought, the forbs of two *Chenopodium* species had a lower seed germination rate compared with *Salosla collina*, an annual plant (Kinugasa et al., 2016). Light can significantly reduce the seed germination rate of perennial grasses regardless of temperature and water conditions (Hu et al., 2013). Several studies have investigated seed germination under different environmental factors, but few have examined how light and temperature and their interaction affect the seed germination percentage, germination time, and germinative force of different plant functional groups.

The grasslands in northern China support animal husbandry, yet these grasslands are sensitive to changes in climate and land use patterns (Zhang et al., 2020; Wang et al., 2020a; Wang et al., 2021). There is thus a need to explore the effects of different temperature and light conditions on seed germination of different functional groups. Here, we conducted temperature and light treatment experiments on seeds of typical grassland plants in northern China, and raised the following questions: (1) How do three plant functional groups respond to temperature and light for seed germination, and (2) and whether there was an interaction between temperature and light on seed germination.

## Materials and Methods

### Study site and materials

The seeds of this study were collected from a temperate steppe located in Duolun County (42°02′N, 116°17′E, 1,324 m a.s.l), Inner Mongolia, Northern China. The long-term mean annual precipitation of the area is 383 mm, and approximately 90% of the annual precipitation falls during the growing season (May to October). The mean annual air temperature is 2.1 °C. The maximum monthly mean temperature (18.9 °C) occurs in July. January is the coldest month with an average temperature of −17.5 °C. The annual accumulated temperature is 1,600–3,200 °C. The plant community of the grassland ecosystem primarily consists of perennial forbs and grasses; annuals and biennials are also common (Sagar et al., 2019; Miao et al., 2020; Wang et al., 2020a).

The seeds of more than 600 native mature plant individuals from 16 common species were collected in semi-arid grassland from September to October 2017. These species belong to the three main functional groups: perennial forbs (PF), perennial grasses (PG), and annuals and biennials (AB). There were nine PF species (*Artemisia frigida, Taraxacum mongolicum, Potentilla tanacetifolia, Potentilla bifurca, Lespedeza davurica, Medicago ruthenica, Plantago asiatica, Allium tenuissimum* L., and *Thalictrum petaloideum*), four PG species (*Stipa krylovii, Agropyron cristatum, Pennisetum centrasiaticum*, and *Leymus chinensis*) and three AB species (*Artemisia scoparia, Chamaerhodos erecta*, and *Dontostemon dentatus*) (Zhong et al., 2019; Miao et al., 2020).

### Seed germination

The seeds were dried and then preserved in the dark at natural temperature until April 2018 for germination experiments. Germination experiments were conducted in 10 cm diameter Petri dishes. This experiment used a factorial design with two factors: light (photoperiod, darkness) and temperature (low temperature, high temperature), which were combined into four different treatments: high temperature/photoperiod (20 °C, 12 h light/12 h dark), low temperature/photoperiod (4 °C, 12 h light/12 h dark), high temperature/darkness (20 °C, 24 h dark), and low temperature/darkness (4 °C, 24 h dark). There were three replicates for each treatment. The different photoperiods were used to simulate the availability of light, darkness is to simulate the expected changes due to nitrogen deposition promotes plant individual growth and litter increase under climate change, which leads to prolongation of dark environment (Hoyle et al., 2014; Chen et al., 2019). A total of 4 °C was used to simulate the snow-melting field temperature in winter (spring) after seed dispersal, and 20 °C was used to simulate the optimal germination temperature of local seeds (Hoyle et al., 2014; Zhang, 2018; Wang et al., 2020b).

First, 192 Petri dishes (16 species × 4 treatments × 3 replicates) were selected for disinfection, a layer of filter paper was placed in each Petri dish. A total of 20 seeds were evenly distributed in each dish and moistened with a spray bottle. Finally, the Petri dishes were placed in different incubators for the germination experiment. Water was added daily for 60 days to keep the Petri dish filter paper moist. Radicle emergence was used as the criterion for germination, and germinating seeds were immediately removed to reduce the disturbance on other seeds (Lai et al., 2019).

### Statistical analysis

Germination was measured using four indices: final germination percentage (FGP), germinative force (GF), germination duration (GD), and germination start (GS):

FGP is the percentage of germinated seeds to tested seeds (Lai et al., 2019; Zhang et al., 2020); GF is the percentage of seed germination at peak to tested seeds. GF measures the speed and uniformity at which seeds germinate. GF and FGP are the main indexes for measuring the quality of seeds (Zhou et al., 2020). GD is the number of days from germination of the first seed to germination of the last seed (Bu et al., 2008); GS is the number of days from the start of the experiment to the germination of the first seed (Chen et al., 2019).

Using data of the 16 species, generalized linear models (GLM) were used to test the effects of temperature and light and their interaction on seed germination of each plant functional group. The sample sizes of PF, PG and AB in each treatment were 27, 12, 9, respectively. F-tests were conducted to evaluate whether GLM predictors explained a significant fraction of the total deviance or not. Tukey’s honestly significant difference (HSD) test was used to evaluate significant differences among multiple treatments based on ANOVA results. Means (±SE) of non-transformed data were calculated and shown in figures. Spearman correlation method was used to determine the correlations among FGP, GF, GD, and GS. All statistical analyses were performed using R software (R Core Team, 2022), and the threshold for statistical significance was *P* < 0.05.

## Results

### Seed final germination proportion

**Figure 1:**
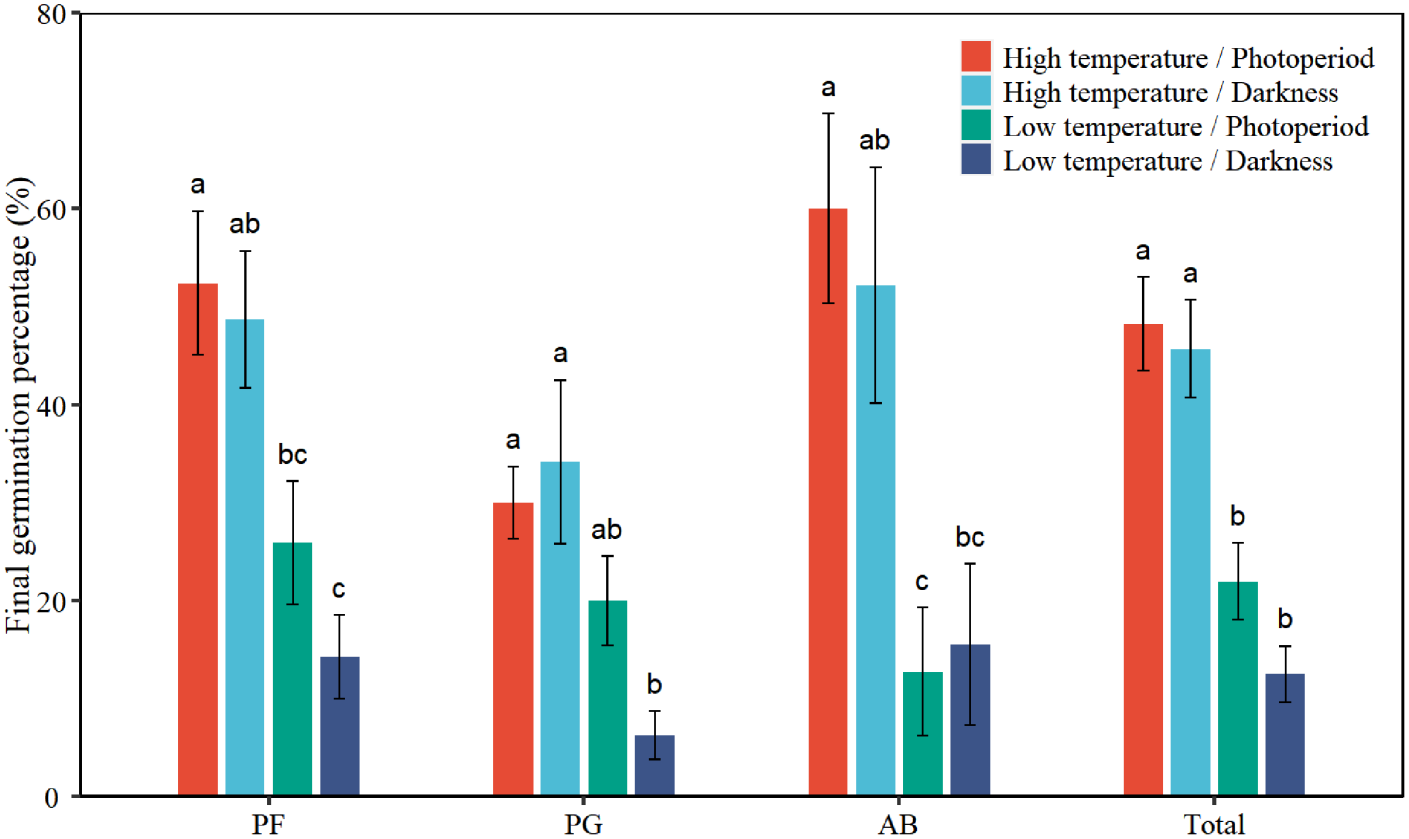
Effects of temperature (high temperature, low temperature) and light (photoperiod, darkness) on seed final germination percentage of total species (Total), perennial forbs (PF), perennial grasses (PG), and annuals and biennials (AB). Error bars indicate the standard error of three replicates. The different letters over the bars represent significant difference among the four treatments based on Tukey’s honestly significant difference test (*P* < 0.05).

Two-way ANOVA indicated that plant functional groups presented a statistically different response in FGP (Table 1, *P* < 0.05). The mean FGP of PG was 22.6%, which was the lowest among the three plant functional groups (Fig. 1). Low temperature significantly inhibited the FGP of total seeds by 29.7% (*P* < 0.001, absolute change, Table 2). Darkness had no significant effect on FPG. There was no interactive effect between temperature and light on the FGP of total seeds. Low temperature significantly inhibited the FGP of PF, PG, and AB by 30.5%, 19.0%, and 41.9%, respectively (Table 2, Fig. 1). The interactions of temperature and light had a significant effect on the FGP of PG (*P* = 0.024). Under photoperiod conditions, low temperature decreased the FGP of PG seeds by 10.0%. Under darkness, low temperature significantly decreased the FGP of PG seeds by 27.9%. Darkness promoted the FGP of PG by 4.2% at high temperature and inhibited the FGP of PG by 13.8% at low temperatures. According to Tukey’s honestly significant difference (HSD) test, the values of FGP of perennial forbs and total species were the highest under the high temperature/photoperiod treatment, and were lowest under low temperature/darkness treatment.

**Table 1:** The effects of light, temperature and plant functional group (PFG) on final germination percentage (FGP), germinative force (GF), germination duration (GD) and germination start (GS) based on generalized linear model analyses.

	FGP		GF		GD		GS	
	F	P	F	P	F	P	F	P
Light	1.895	0.170	4.978	**0.027**	1.639	0.202	1.506	0.221
Temperature	48.237	**<0.001**	68.338	**<0.001**	4.903	**0.028**	378.742	**<0.001**
PFG	3.698	**0.027**	7.837	**<0.001**	0.487	0.616	0.808	0.448
Light × Temperature	1.698	0.194	2.831	0.094	0.931	0.336	0.379	0.539
Light × PFG	0.109	0.897	0.292	0.747	2.405	0.093	0.068	0.935
Temperature × PFG	0.829	0.438	2.123	0.123	0.705	0.495	1.141	0.322
Light × Temperature × PFG	0.962	0.384	0.375	0.688	0.954	0.387	1.777	0.172

**Note:**

Significant effects (*P* < 0.05) are in bold.

**Table 2:** The effects of light and temperature on final germination percentage (FGP), germinative force (GF), germination duration (GD) and germination start (GS) of perennial forbs (PF), perennial grasses (PG), annuals and biennials (AB) and total specie (Total) based on generalized linear model analyses.

		FGP		GF		GD		GS	
		F	*P*	F	*P*	F	*P*	F	*P*
PF	Light	1.355	0.247	3.960	**0.049**	0.261	0.611	0.653	0.421
	Temperature	21.943	**<0.001**	24.260	**<0.001**	3.616	0.060	194.280	**<0.001**
	Light × Temperature	0.879	0.351	1.734	0.191	1.277	0.261	1.621	0.206
PG	Light	0.863	0.358	0.929	0.340	10.645	**0.002**	**0.986**	0.326
	Temperature	13.879	**<0.001**	30.181	**<0.001**	4.047	**0.050**	**123.118**	**<0.001**
	Light × Temperature	5.042	**0.030**	4.080	**0.050**	2.235	0.142	0.154	0.697
AB	Light	0.059	0.810	0.386	0.539	0.318	0.577	0.115	0.737
	Temperature	17.419	**0.000**	27.542	**<0.001**	0.001	0.977	67.933	**<0.001**
	Light × Temperature	0.259	0.614	0.000	0.996	0.390	0.537	1.853	0.183
Total	Light	1.834	0.177	4.381	**0.038**	1.566	0.212	1.450	0.230
	Temperature	46.694	**<0.001**	60.134	**<0.001**	4.683	**0.032**	**364.650**	**<0.001**
	Light × Temperature	1.641	0.202	2.501	0.115	0.889	0.347	0.365	0.547

**Note:**

Significant effects (*P* < 0.05) are in bold.

### Seed germinative force

**Figure 2:**
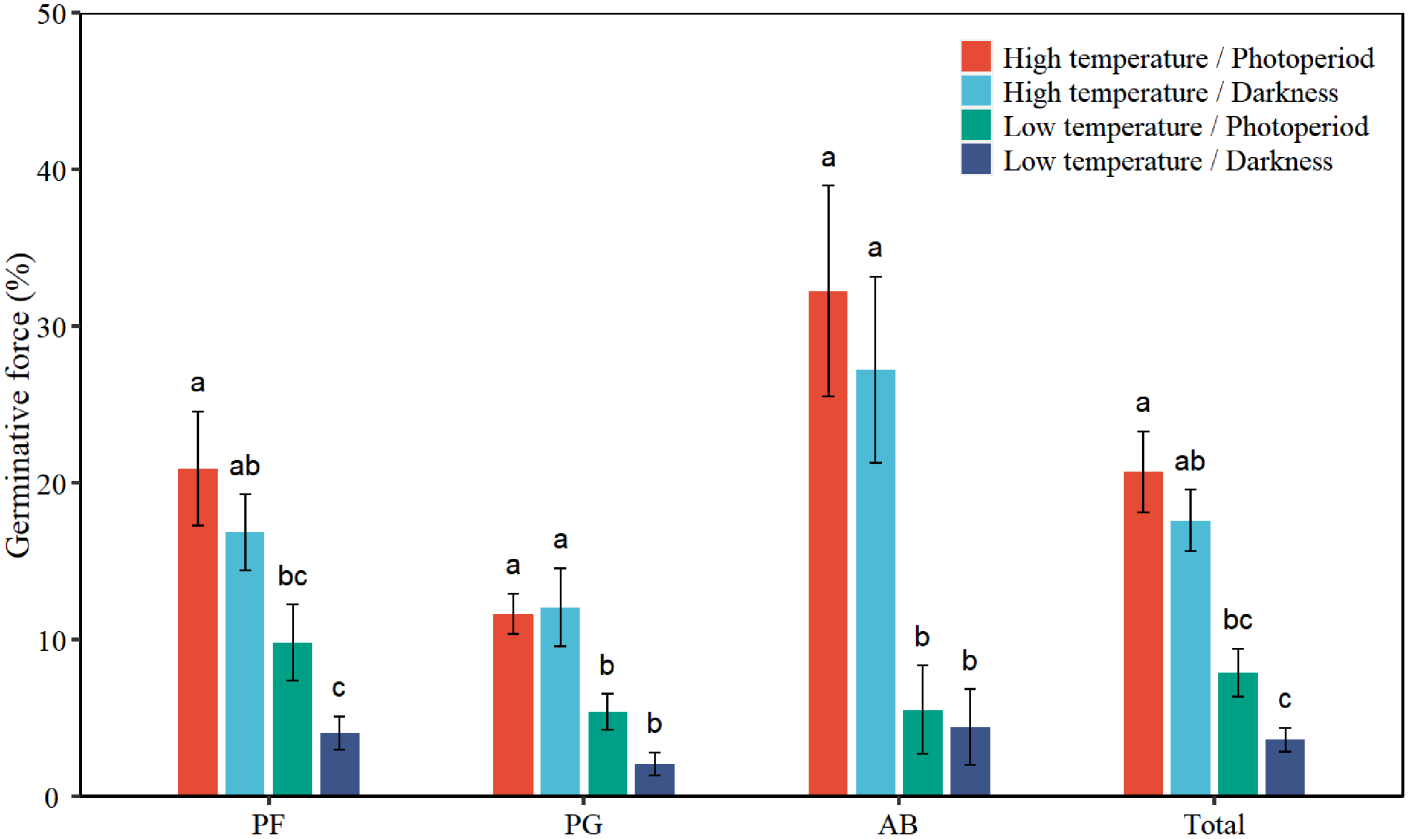
Effects of temperature (high temperature, low temperature) and light (photoperiod, darkness) on seed germinative force of total species (Total), perennial forbs (PF), perennial grasses (PG), and annuals and biennials (AB). Error bars indicate the standard error of three replicates. The different letters over the bars represent significant difference among the four treatments based on Tukey’s honestly significant difference tests (*P* < 0.05).

There were statistical differences among plant functional groups in FGP (Table 1, Fig. 2). Low temperature and darkness significantly decreased the GF of total seeds by 13.4% and 3.7%, respectively. There was no interaction between the effects of temperature and light on the GF of total seeds. Low temperature significantly decreased the GF of PF, PG, and AB by 11.9%, 8.1%, and 24.7%, respectively (Table 2, Fig. 2). Darkness significantly reduced the GF of PF by 4.9%. The interaction between temperature and light significantly affected the GF of PG (*P* = 0.043, Table 2). Under photoperiod conditions, low temperature significantly reduced the GF of PG seeds by 6.3%. Under darkness, low temperature significantly decreased the GF of PG seeds by 10%. According to Tukey’s honestly significant difference (HSD) test, the values of GF of perennial forbs and total species were the highest under the high temperature/photoperiod treatment, and were lowest under low temperature/darkness treatment.

### Seed germination duration

**Figure 3:**
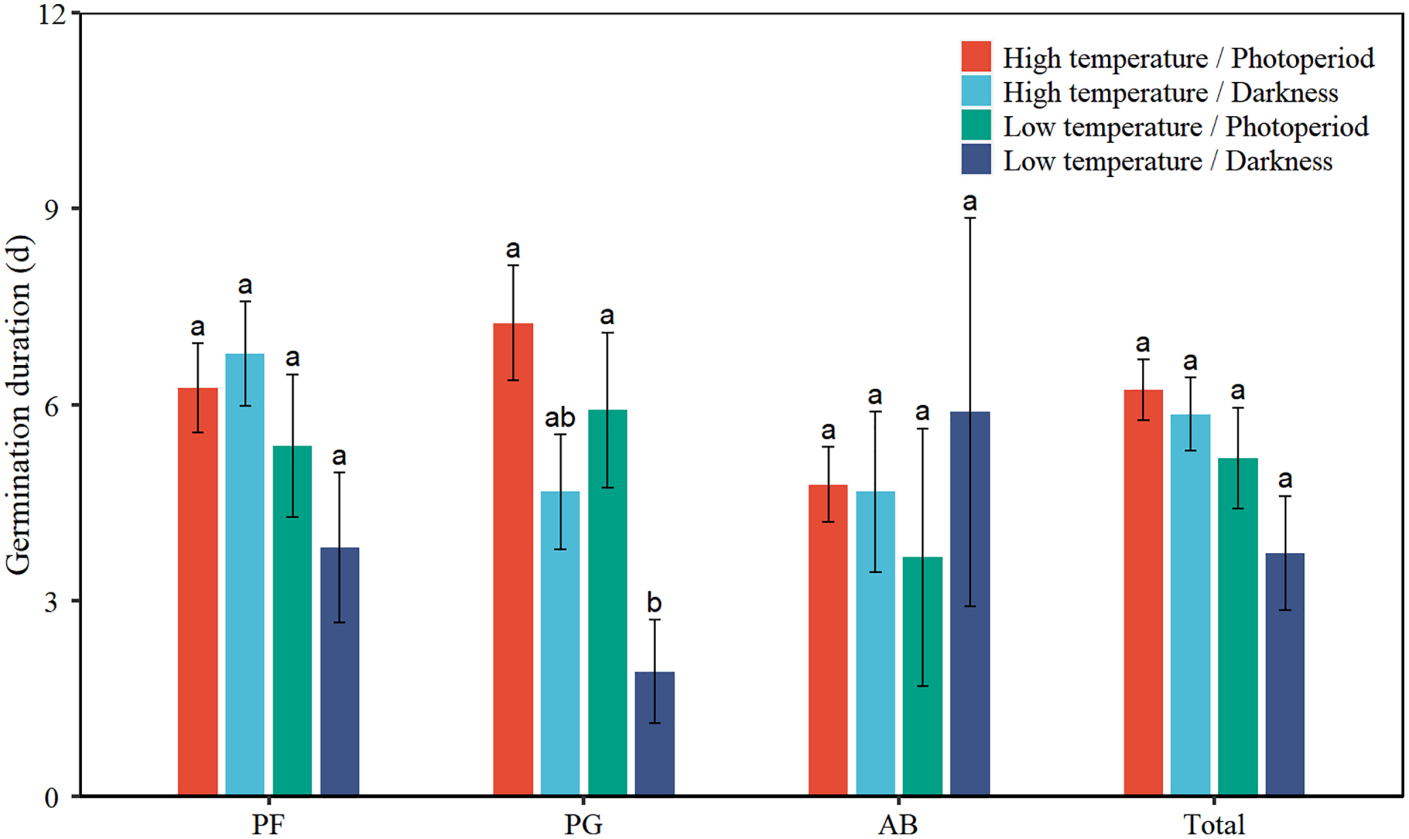
Effects of temperature (high temperature, low temperature) and light (photoperiod, darkness) on seed germination duration of total species (Total), perennial forbs (PF), perennial grasses (PG), and annuals and biennials (AB). Error bars indicate the standard error of three replicates. The different letters over the bars represent significant difference among the four treatments based on Tukey’s honestly significant difference tests (*P* < 0.05).

Low temperature significantly decreased the GD of total seeds by 1.6 days (Table 2, Fig. 3). Low temperature significantly reduced the GD of PG by 2.0 days. Darkness significantly reduced the GD of PG by 3.3 days. There was no interaction effect between temperature and light on the GD of total seeds and the three functional groups.

### Seed germination start

**Figure 4:**
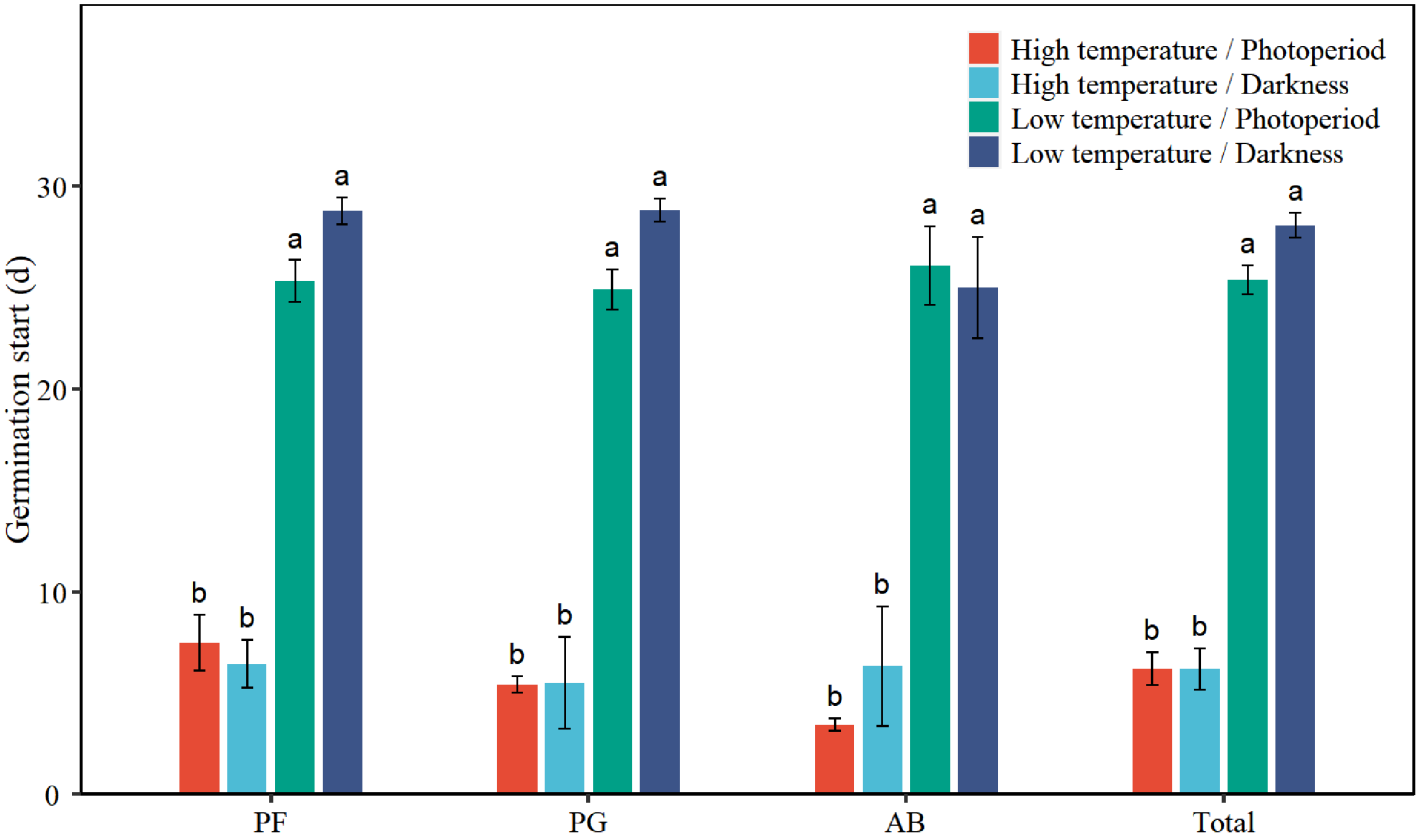
Effects of temperature (high temperature, low temperature) and light (photoperiod, darkness) on seed germination start of total species (Total), perennial forbs (PF), perennial grasses (PG), and annuals and biennials (AB). Error bars indicate the standard error of three replicates. The different letters over the bars represent significant difference among four treatments based on Tukey’s honestly significant difference tests (*P* < 0.05).

Low temperature significantly prolonged the GS of total seeds by 19.8 days (Table 1, Fig. 4). Low temperature significantly increased the GS of PF, PG, and AB by 18.9, 21.0, and 20.7 days, respectively (Table 2, Fig. 4). Darkness had no significant effect on the GS of seed germination. There was no significant interaction effect between temperature and light on the GS of total seeds and different functional groups.

### Relationships between germination indexes

**Figure 5:**
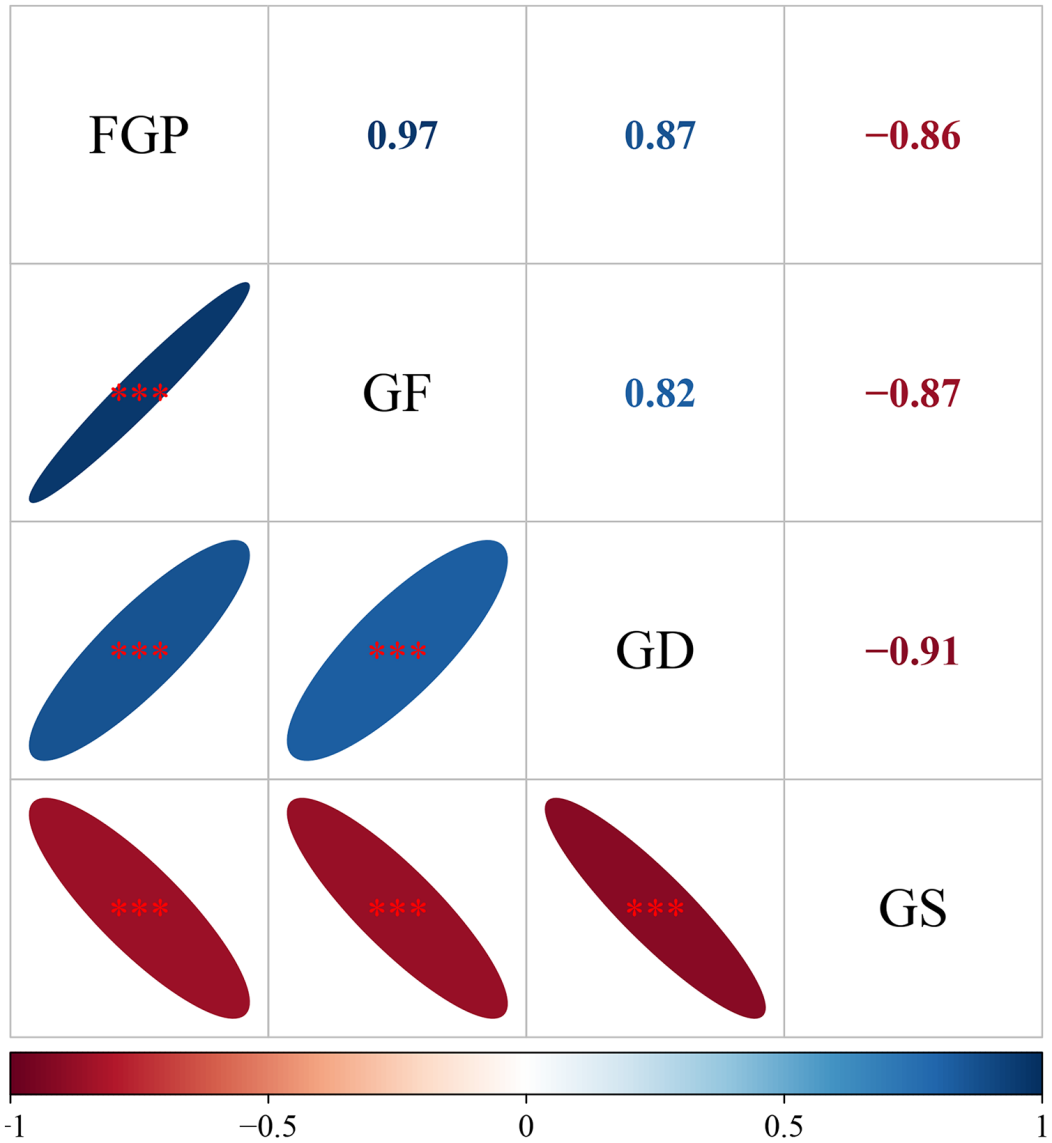
The relationship among final germination percentage, germinative force, germination duration, and germination start of total species. Each data point represents the mean value of each species across the four treatments. ***P < 0.01, **P < 0.01, *P < 0.05.

The mean FGP, GF, and GD of all species were negatively correlated with the mean GS (Fig. 5). There was a pairwise positive correlation among the average FGP, GF, and GD of all species.

## Discussion

### Effect of temperature on seed germination

Seed germination was sensitive to environmental conditions, and excessively high or low temperatures were not conducive to seed germination (Hadi et al., 2018; Chen et al., 2019; Zhang et al., 2020). In this study, low temperature significantly decreased the final germination percentage and germination force of all seeds. This is consistent with the results of previous studies indicating that low temperatures could significantly inhibit seed germination (Lai et al., 2019). In this experiment, 4 °C was used to simulate the snow-melting field temperature in winter (early spring) after seed dispersal, and 20 °C was used to simulate the optimal germination temperature of local seeds (Hoyle et al., 2014). The optimal temperature for seed germination was closely related to the maternal habitat (Liu, Qi & Shu, 2004). Suboptimal temperatures could affect the activity of a series of cytoplasmic enzymes and cell membrane permeability, which in turn affected the process of seed germination (Finch-Savage & Leubner-Metzger, 2006; Penfield, 2017). The low temperature treatment might lead to decreases in the enzyme activity and metabolism in seeds and thereby inhibit seed germination. Low temperature significantly shortened the germination duration of total seeds. The accumulated cold temperature before seed germination could induce or accelerate seed development and thus shorten the germination duration (Chen et al., 2019). Low temperature could also restrict seed germination, as the time required for the germination of the tested seeds increase. Correlation analysis indicated that the duration of seed germination increased as the final germination percentage and germinative force increased (Fig. 5). Decreases in the germination duration indicated that some early germinating species might gain a competitive advantage through increased access to resources (Wang et al., 2020b).

Low temperature had different effects on seed germination percentage and germinative force of different functional groups. Compared with perennial forbs and perennial grasses, seeds of annual and biennials were more sensitive to low temperature. This finding was consistent with the results of previous experiments showed that low temperature reduced seed germination of annual plants and induced dormancy (Zhang et al., 2015). Short-lived plants had more dormant seeds than long-lived plants as well as more requirements for their seeds to germinate (Bu et al., 2008). Compared with perennials, annual plants only produced seeds once in their lifetime and were more dependent on the environment in which seeds germinate. Under harsh environmental conditions, plants had two germination strategies: adventurous germination or dormancy (Greenberg, Smith & Levey, 2001). Once an annual plant failed to germinate, it lost the seed genotype that does not germinate, and thus the annual plant goes into dormancy to forego the risk of germination (Bu et al., 2008). The final germination percentage was the lowest for perennial grasses. This might be explained by the fact that perennial species did not depend on successful germination in any year, nor on the establishment of a persistent seed bank, because they could survive for a long time through vegetative growth (Wesche et al., 2006). Previous study had shown that dominant perennial plants, such as *Agropyron cristatum* and *Stipa gobiaa*, did not produce new seedlings for many years (Wesche et al., 2006). Seeds buried in the soil sense temperature changes and selected suitable times to initiate their life cycle (Chen et al., 2019). Therefore, short-term changes in the plant community might stem from changes in annual and biennial plants (Zeng et al., 2016; Anniwaer et al., 2020). In addition, the final germination percentage of total seeds in this experiment was low, this might stem partly from the fact that seeds were stored at room temperature after being collected from the field, which reduced seed vigor (Liu, Qi & Shu, 2004; Shen et al., 2008). Some studies had shown that seed vigor was better maintained when seeds were refrigerated (Liu, Qi & Shu, 2004). The responses of seed germination of perennial and annual plants to low temperature differed, indicating that the various germination strategies employed by different plant functional groups might affect the community structure.

Global warming will likely result in shorter winters and the melting of snow (Walck et al., 2011). Reductions in snow cover resulted in colder soil and deeper soil frosts; this could cause germinated seedlings to die or seeds to go back into dormancy, which leaved more seeds in the soil seed bank (Walck et al., 2011). In this study, it was impossible to identify the effect of fluctuating temperatures on seed germination (Shen et al., 2008). Seed of some species could come out of dormancy only after they were exposed to fluctuating temperatures (Benech-Arnold et al., 2000). Therefore, it is necessary to further explore the effects of changes in plant seed functional groups on plant community structure under different temperature fluctuations.

### Effect of light on seed germination

Light was a key environmental factor affecting seed germination (Finch-Savage & Leubner-Metzger, 2006). After seed maturity and shedding, seeds might be distributed in different environments on the soil surface. For seeds in soil, the spectral composition and irradiance of light were important signals that can indicate the suitability of environmental conditions (Gu et al., 2005). Differences in illumination might induce the dormancy or germination of plant seeds (Gresta et al., 2010). The dark conditions used in this study had also been examined in previous studies (Hoyle et al., 2014; Chen et al., 2019). Increased litter, mainly due to nitrogen deposition, limited the availability of light and increased the possibility that plant seeds would be covered when they left the parent plant (Jensen & Gutekunst, 2003). Darkness significantly reduced seed germinative force, which might stem from the mechanism by photosensitivity (Gresta et al., 2010). The photosensitive properties of plants prevented seeds from being established in shaded environments covered with litter or trees; consequently, appropriate sites needed to be identified to promote the establishment of seedlings after germination (El-Keblawy, 2017). Seeds could use light to detect the distance from the ground and thus identified suitable sites to promote the establishment of seedlings after germination (Flores, Gonzalez-Salvatierra & Jurado, 2016). Darkness significantly reduced the germinative force of perennial forbs, but had no significant effect on perennial grasses or annual and biennial plants. Previous studies had shown that two *Chenopodium* plants had low seed final germination percentage under the combined action of light and temperature (Kinugasa et al., 2016). These differences led to variation in the germination time and space of different species and functional groups in semiarid grassland community. Plant functional groups had evolved different mechanisms to cope with environmental resource scarcity.

The decrease in seed germination under darkness might protect established plant seedlings from limitations in light resources; canopy space was an important factor limiting the establishment of seedlings (Olff et al., 1994). The increase in plant litter promoted by nitrogen deposition increased the amount of surface cover and created a dark environment that affected seed germination (Jensen & Gutekunst, 2003; Zhang, Wang & Wan, 2019). For some plant seeds that are buried under leaf litter, the need for light to induce germination during burial may prevent germination (Schutz & Rave, 1999). Therefore, light competition could limit the richness of plant species through seed germination (Yang et al., 2011). Under environmental conditions that were not conducive to germination, seeds remain in a dormant state until conditions were suitable (Hu et al., 2013). These results indicated that the dark conditions caused by the litter would affect the process of seed germination, and the light limitation of litter could be reduced by proper grazing and mowing in the future to promote plant establishment (Yuan, Liang & Zhang, 2016).

### Interaction effect of temperature and light on seed germination

Environmental factors such as temperature and light were key factors affecting seed germination (Gao et al., 2012). Seed germination could only respond to specific combinations of environmental factors (Yi et al., 2019), and adverse temperature and light conditions, individually or in combination, might prevent the germination of newly shed seeds (Schutz & Rave, 1999). In this study, low temperature and darkness had a significant interaction effect on the final germination percentage and germinative force of perennial grass. Darkness intensified the inhibitory effect of low temperature on seed germination of perennial grass. Seed final germination percentage was the lowest under the combined action of darkness and low temperature, and this interaction between light and temperature also affected germinative force of perennial grass (Wu et al., 2016). These observations indicated that interactions among different environmental factors could affect seed germination, and differences were observed among the different plant functional groups (Wu et al., 2016; Chen et al., 2019; Yi et al., 2019). Johnson’s experiment (2012) showed that the interaction between light and temperature affected seed germination by demonstrating that higher temperatures were required for seeds to germinate in the presence of light (Johnson & Kane, 2012). Furthermore, in some plants with strong photosensitivity, seed germination was mediated by temperature-controlled phytochromes (Yang et al., 1995). Plants had evolved strategies that involve both predicting germination and optimizing their adaptability, wherein some seeds were allowed to germinate in the current environment while others remain dormant, thus hedging their bets on unpredictable conditions that were not conducive to seedling establishment (Yi et al., 2019).

The findings of this study suggested that low temperature significantly inhibited seed final germination percentage, especially that of annual and biennial plants. This effect had also been observed in adult plants in terrestrial ecosystems. Annuals were more sensitive to temperature changes than perennials, and their growth would be promoted by changes in temperature (Zhou et al., 2011). Many annual and biennial plants had a better bet-hedging strategy for completing their life cycle earlier under suitable conditions, which provided an advantage in resource competition (Gremer & Venable, 2014; Zhang et al., 2020). The results of seed germination at the functional group level were consistent with those found at the plant community level, indicating that the response of seed germination to environmental changes could explain community changes. Under multi-factor climate change, the responses of seed germination of the plant community would be complex. Seed germination was a key stage in plant life history, but it was only the first step, and there was still a lot of uncertainty about how the structure of plant community might change. In addition, to verify the long-term effects of climate change on plant community structure, multi-year sampling and increasing sample numbers are required, while focusing on whether seed germination status is consistent with the response of adult plant communities to climate change.

## Conclusion

We found that low temperature had significant negative effects on seed final germination percentage, germinative force, germination duration, and germination start at both the community level and the functional group level. The negative effects of low temperature on the final germination percentage and germinative force were higher for annuals and biennials than for other plant functional groups. Perennial grasses were affected by the interaction between low temperature and darkness. Darkness strengthened the inhibitory effect of low temperature on seed final germination percentage and germination force of perennial grasses. The changes in community structure caused by the diverse response of different functional groups affected the original ecological services provided by ecosystems. The responses of seed germination of plant functional groups to changes in the environmental conditions in semiarid grasslands require further exploration for explaining the responses and changes in the ecological function of plant communities under future climate change.

## Supplemental Information

10.7717/peerj.14485/supp-1 Supplemental Information 1 Raw Data.

## Additional Information and Declarations

### Data Availability

The following information was supplied regarding data availability: The raw data is available as a Supplemental File.

## References

[ref-1] Anniwaer A, Su YG, Zhou XB, Zhang YM (2020). Impacts of snow on seed germination are independent of seed traits and plant ecological characteristics in a temperate desert of Central Asia. Journal of Arid Land.

[ref-2] Baskin CC, Baskin JM (1998). Ecology, biogeography, and evolution of dormancy and germination-Introduction. Seeds.

[ref-3] Benech-Arnold RL, Sanchez RA, Forcella F, Kruk BC, Ghersa CM (2000). Environmental control of dormancy in weed seed banks in soil. Field Crops Research.

[ref-4] Bu HY, Du GZ, Chen XL, Xu XL, Liu K, Wen SJ (2008). Community-wide germination strategies in an alpine meadow on the eastern Qinghai-Tibet plateau: phylogenetic and life-history correlates. Plant Ecology.

[ref-5] Chen Y, Cao Q, Li D, Liu H, Zhang D (2019). Effects of temperature and light on seed germination of ephemeral plants in the Gurbantünggüt Desert, China: implications for vegetation restoration. Journal of Arid Land.

[ref-6] Chen J, Luo Y, Chen Y, Felton AJ, Hopping KA, Wang R-W, Niu S, Cheng X, Zhang Y, Cao J, Olesen Jørgen E, Andersen MN, Jørgensen U (2020). Plants with lengthened phenophases increase their dominance under warming in an alpine plant community. Science of the Total Environment.

[ref-7] Dürr C, Dickie JB, Yang X-Y, Pritchard HW (2015). Ranges of critical temperature and water potential values for the germination of species worldwide: contribution to a seed trait database. Agricultural and Forest Meteorology.

[ref-8] El-Keblawy A (2017). Germination response to light and temperature in eight annual grasses from disturbed and natural habitats of an arid Arabian desert. Journal of Arid Environments.

[ref-9] Finch-Savage WE, Leubner-Metzger G (2006). Seed dormancy and the control of germination. New Phytologist.

[ref-10] Flores J, Gonzalez-Salvatierra C, Jurado E (2016). Effect of light on seed germination and seedling shape of succulent species from Mexico. Journal of Plant Ecology.

[ref-11] Gao S, Wang J, Zhang Z, Dong G, Guo J (2012). Seed production, mass, germinability, and subsequent seedling growth responses to parental warming environment in *Leymus chinensis*. Crop & Pasture Science.

[ref-12] Greenberg CH, Smith LM, Levey DJ (2001). Fruit fate, seed germination and growth of an invasive vine—an experimental test of ‘sit and wait’ strategy. Biological Invasions.

[ref-13] Gremer JR, Venable DL (2014). Bet hedging in desert winter annual plants: optimal germination strategies in a variable environment. Ecology Letters.

[ref-14] Gresta F, Cristaudo A, Onofri A, Restuccia A, Avola G (2010). Germination response of four pasture species to temperature, light, and post-harvest period. Plant Biosystems.

[ref-15] Gu AL, Yi J, Roman H, Zbigniew B (2005). Effects of low-temperatures on seed germination of *Leymus chinensis* and *Pascopyrum smithii*. Grassland of China.

[ref-16] Hadi SMS, Ahmed MZ, Hameed A, Khan MA, Gul B (2018). Seed germination and seedling growth responses of toothbrush tree (*Salvadora persica Linn*.) to different interacting abiotic stresses. Flora.

[ref-17] Hoyle GL, Cordiner H, Good RB, Nicotra AB (2014). Effects of reduced winter duration on seed dormancy and germination in six populations of the alpine herb *Aciphyllya glacialis* (Apiaceae). Conservation Physiology.

[ref-18] Hu XW, Zhou ZQ, Li TS, Wu YP, Wang YR (2013). Environmental factors controlling seed germination and seedling recruitment of *Stipa bungeana* on the Loess Plateau of northwestern China. Ecological Research.

[ref-19] Jensen K, Gutekunst K (2003). Effects of litter on establishment of grassland plant species: the role of seed size and successional status. Basic and Applied Ecology.

[ref-20] Johnson TR, Kane ME (2012). Effects of temperature and light on germination and early seedling development of the pine pink orchid (*Bletia purpurea*). Plant Species Biology.

[ref-21] Kinugasa T, Hozumi Y, Nishizima H, Ishitobi A, Miyawaki M (2016). Germination characteristics of early successional annual species after severe drought in the Mongolian steppe. Journal of Arid Environments.

[ref-22] Lai L, Chen L, Zheng M, Jiang L, Zhou J, Zheng Y, Shimizu H (2019). Seed germination and seedling growth of five desert plants and their relevance to vegetation restoration. Ecology and Evolution.

[ref-23] Li ZJ, Liang MW, Li ZY, Mariotte P, Tong XZ, Zhang JH, Dong L, Zheng Y, Ma WH, Zhao LQ, Wang LX, Wen L, Tuvshintogtokh I, Gornish ES, Dang ZH, Liang CZ, Li FY (2021). Plant functional groups mediate effects of climate and soil factors on species richness and community biomass in grasslands of Mongolian Plateau. Journal of Plant Ecology.

[ref-24] Li W, Zhao J, Epstein HE, Jing G, Cheng J, Du G (2017). Community-level trait responses and intra-specific trait variability play important roles in driving community productivity in an alpine meadow on the Tibetan Plateau. Journal of Plant Ecology.

[ref-25] Liu GS, Qi DM, Shu QY (2004). Seed germination characteristics in the perennial grass species *Leymus chinensis*. Seed Science and Technology.

[ref-26] Miao Y, Xuan J, Zhang Z, Chen A, Shen H, Liu Y (2020). Effects of artificial irrigation on grasslands production in different types of Inner-Mongolia steppe. Pakistan Journal of Botany.

[ref-27] Miransari M, Smith DL (2014). Plant hormones and seed germination. Environmental and Experimental Botany.

[ref-28] Olff H, Pegtel DM, Vangroenendael JM, Bakker JP (1994). Germination strategies during grassland succession. Journal of Ecology.

[ref-29] Penfield S (2017). Seed biology—from lab to field. Journal of Experimental Botany.

[ref-57] R Core Team (2022). A language and environment for statistical computing.

[ref-30] Ronnenberg K, Wesche K, Pietsch M, Hensen I (2007). Seed germination of five mountain steppe species of Central Asia. Journal of Arid Environments.

[ref-31] Sagar R, Li GY, Singh JS, Wan S (2019). Carbon fluxes and species diversity in grazed and fenced typical steppe grassland of Inner Mongolia, China. Journal of Plant Ecology.

[ref-32] Schütz W, Rave G (1999). The effect of cold stratification and light on the seed germination of temperate sedges (Carex) from various habitats and implications for regenerative strategies. Plant Ecology.

[ref-33] Seo M, Nambara E, Choi G, Yamaguchi S (2009). Interaction of light and hormone signals in germinating seeds. Plant Molecular Biology.

[ref-34] Shahverdi MA, Omidi H, Mosanaiey H, Pessarakli M, Mousavi SE, Ghasemzadeh M (2019). Effects of light and temperature treatments on germination and physiological traits of stevia seedling (*Stevia rebuadiana* Bertoni). Journal of Plant Nutrition.

[ref-35] Shen JB, Xu LY, Jin XQ, Chen JH, Lu HF (2008). Effect of temperature regime on germination of seed of perennial ryegrass (*Lolium perenne*). Grass and Forage Science.

[ref-36] Su L, Yang Y, Li X, Wang D, Liu Y, Liu Y, Yang Z, Li M (2018). Increasing plant diversity and forb ratio during the revegetation processes of trampled areas and trails enhances soil infiltration. Land Degradation & Development.

[ref-37] Tabatabaei SA (2015). The changes of germination characteristics and enzyme activity of barley seeds under accelerated aging. Cercetari Agronomice in Moldova.

[ref-38] Tobe K, Zhang LP, Omasa K (2005). Seed germination and seedling emergence of three annuals growing on desert sand dunes in China. Annals of Botany.

[ref-39] Walck JL, Hidayati SN, Dixon KW, Thompson K, Poschlod P (2011). Climate change and plant regeneration from seed. Global Change Biology.

[ref-40] Wang D, Chi Z, Yue B, Huang X, Zhao J, Song H, Yang Z, Miao R, Liu Y, Zhang Y, Miao Y, Han S, Liu Y (2020a). Effects of mowing and nitrogen addition on the ecosystem C and N pools in a temperate steppe: a case study from northern China. CATENA.

[ref-41] Wang D, Huang X, Qiao N, Geng Q, Liu Y, Song H, Yang Z, Liu C, Wang G (2021). Effects of mowing and fertilization on soil quality in a semiarid grassland of North China. Land Degradation & Development.

[ref-42] Wang X, Niu B, Zhang X, He Y, Shi P, Miao Y, Cao Y, Li M, Wang Z (2020b). Seed germination in alpine meadow steppe plants from Central Tibet in response to experimental warming. Sustainability.

[ref-43] Wang D, Zhu YJ, Wu GL, Feng J (2014). Seedling performance within eight different seed-size alpine forbs under experimentation with irradiance and nutrient gradients. Pakistan Journal of Botany.

[ref-44] Wesche K, Pietsch M, Ronnenberg K, Undrakh R, Hensen I (2006). Germination of fresh and frost-treated seeds from dry Central Asian steppes. Seed Science Research.

[ref-45] Wu YP, Chen F, Hu XW, Baskin CC, Baskin JM (2016). Alleviation of salinity stress on germination of *Leymus chinensis* seeds by plant growth regulators and nitrogenous compounds under contrasting light/dark conditions. Grass and Forage Science.

[ref-46] Yang HJ, Li Y, Wu MY, Zhang Z, Li LH, Wan SQ (2011). Plant community responses to nitrogen addition and increased precipitation: the importance of water availability and species traits. Global Change Biology.

[ref-47] Yang YY, Nagatani A, Zhao YJ, Kang BJ, Kendrick RE, Kamiya Y (1995). Effects of gibberellins on seed germination of phytochrome-deficient mutants of *Arabidopsis thaliana*. Plant and Cell Physiology.

[ref-48] Yi F, Wang Z, Baskin CC, Baskin JM, Ye R, Sun H, Zhang Y, Ye X, Liu G, Yang X, Huang Z (2019). Seed germination responses to seasonal temperature and drought stress are species-specific but not related to seed size in a desert steppe: implications for effect of climate change on community structure. Ecology and Evolution.

[ref-49] Yuan J, Liang D, Zhang S (2016). Litter and its interaction with standing vegetation affect seedling recruitment in Tibetan alpine grasslands. Plant Ecology & Diversity.

[ref-50] Zeng Y, Liu T, Zhou X-B, Sun Q-M, Han Z-Q, Liu K (2016). Effects of climate change on plant composition and diversity in the Gurbantünggüt Desert of northwestern China. Ecological Research.

[ref-51] Zeng Y, Wang Y, Nan Z, Wei D, Chen S, Li B (2003). Soil seed banks of different grassland types of Alashan arid desert region, Inner Mongolia. Chinese Journal of Applied Ecology.

[ref-59] Zhang Q (2018). Effect of temperature and light on seeds germination in semi-arid grasslands. Journal of Henan University (Natural Science).

[ref-52] Zhang T, Guo R, Gao S, Guo JX, Sun W (2015). Responses of plant community composition and biomass production to warming and nitrogen deposition in a temperate meadow ecosystem. PLOS ONE.

[ref-53] Zhang T, Liu M, Huang X, Hu W, Qiao N, Song H, Zhang B, Zhang R, Yang Z, Liu Y, Miao Y, Han S, Wang D (2020). Direct effects of nitrogen addition on seed germination of eight semi-arid grassland species. Ecology and Evolution.

[ref-54] Zhang A, Wang D, Wan S (2019). Litter addition decreases plant diversity by suppressing seeding in a semiarid grassland, Northern China. Ecology and Evolution.

[ref-58] Zhong MX, Miao Y, Han SJ, Wang D (2019). Nitrogen addition decreases seed germination in a temperate steppe. Ecology and Evolution.

[ref-55] Zhou W, Lu X, Qi C, Yang M (2020). Utilisation of ultrasonic treatment to improve the soil amelioration property of coal fly ash. Journal of Environmental Management.

[ref-56] Zhou X, Zhang Y, Ji X, Downing A, Serpe M (2011). Combined effects of nitrogen deposition and water stress on growth and physiological responses of two annual desert plants in Northwestern China. Environmental and Experimental Botany.

